# Prevalence of sickle cell disease and sickle cell trait among children admitted to Al Fashir Teaching Hospital North Darfur State, Sudan

**DOI:** 10.1186/s13104-019-4682-5

**Published:** 2019-10-16

**Authors:** Mudathir A. Adam, Nassreldeen K. Adam, Babiker A. Mohamed

**Affiliations:** 1 Faculty of Medical Laboratory Science, Al Fashir University, Al Fashir, Sudan; 2 Pathology Department, Faculty of Medicine, Karari University, Khartoum, Sudan

**Keywords:** Sickle cell disease, Hb SS, Hb AS, Hb electrophoresis, Children, Al Fashir

## Abstract

**Objective:**

It is estimated that 50% to 90% of infants born with (SCA) in sub-Saharan Africa die before 5 years old. Northern Darfur State at western Sudan region has a multiethnic population with a high frequency of sickle cell anaemia, but little about it is published. This study aimed to determine the prevalence of sickle cell anaemia among children admitted to Al Fashir Teaching Hospital in Al Fashir, Northern Darfur State, Sudan.

**Results:**

The prevalence of sickle cell disease by haemoglobin electrophoresis among these 400 children patients was 59 (14.8%). Sickle cell trait patients were 11.3% and Sickle cell disease positive patients were 3.5%. Individuals with SCA have consistently low blood Hb concentration, normal MCV and high mean WBC’s. Individuals with sickle cell trait had haematological parameters near to those of normal individuals.

## Introduction

Sickle cell anaemia (SCA) is an autosomal recessive blood disorder lead to the production of red blood cells that appear abnormal. The disease occurs due to a mutation in the haemoglobin-encoding gene [1, 2].

Hb S gene prevalence in at least 40 countries varies between 2 and 30%, resulting in high SCD-related morbidity and mortality. Deaths from SCA complications occur mostly in children under 5 years, adolescents and pregnant women [3–5].

Exceed 75% of the global burden of SCA occurs in sub-Saharan Africa, where health resources insufficiency accompanied by lack of training among health care staffs and the lack of general public awareness contribute to shocking rates of early mortality. True mortality rates are unknown due to the shortage of data from neonatal screening programs, and the absence of prospective natural history studies, [4–6]. It is estimated that 50% to 90% of infants born with (SCA) in sub-Saharan Africa die before 5 years old [6–8].

Archibald reported the first case of sickle cell anaemia in Sudan in 1926, considered the first reported case in Africa [9]. The prevalence rate of sickle cell anaemia in Sudan ranging from 2 to 30.4% [10]. Western Sudan residents represent the highest prevalence of SCA in the Sudanese population. Immigrants from West African tribes, especially Housa, Folani and Bargo, are thought to have brought sickle cell gene to Sudan [11–13].

Sickle cell anaemia is very well documented among Albagara tribes, a study performed by Bayoumi et al. in western Sudan [14]. Also, in a subgroup of Albagara, Messeryia, studies conducted by Ahmed and his colleagues in 1986 showed that, the prevalence of sickle cell disease was 30% [15] and 16% among immigrants from the Blue Nile province and 18% among Nilotic tribes in the south of Sudan in separate study done by Foy et al. [16]. Furthermore, Hb S is also known to be prevalent in the White Nile (1986) and Khartoum states (1972) as documented by Ahmed and his colleagues [15] and Omer et al. [17] respectively.

## Main text

### Methods

#### Time and duration of the study

This was a descriptive cross-sectional hospital-based study, carried out from December 2017 to August 2018 among children 0–18 years old who were admitted to Al Fashir Teaching Hospital, Sudan. The hospital is a 285-bed tertiary care facility, which serves as a referral Centre for North Darfur State. The average all ages patient turnover at the hospital is 150–300 patients per day.

#### Study population

Four hundred children patients at the pediatric ward Al Fashir hospital during the study period were asked to answer a structured questionnaire consisting of socio-demographic data (age, gender, and tribe). The informed consent related volunteers were recruited randomly, any child admitted to the pediatric ward has a chance to be selected a once. Four hundred children parents have consented for their children to participate in this study with a moderate rate of refusal.

#### Blood samples collection

Peripheral venipuncture (under completely aseptic condition) was used to draw 5 ml of blood from each patient. Samples were sent to the laboratory within 5 min, samples were then immediately tested for complete blood count using an automated haematological analyzer, Sysmex Kx 21N (Toa Medical Electronics, Japan) and haemoglobin electrophoresis using MINICAP HEMOGLOBIN capillary zone electrophoresis (CZE) (Sebia, France).

#### Capillary zone electrophoresis

The blood sample was kept at 2–8 °C for several hours to form a sediment. Then centrifuged at 5000 rpm for 5 min, and the plasma was discarded. The sediment was vortexed for 5 s; a sufficient number of reagent cups for analysis in the reagent cup holder was inserted. The level of haemoglobin buffers and wastes were verified. Tests samples and Normal Hb A2 control each were placed into a new haemolysing tube and labelled with the specific barcode for normal Hb A2 controls and tests, then were placed in the appropriate position of the carousel. Results were displayed on the screen and were printed out.

Data (Additional file 1) were analyzed by the statistical software package of social sciences (SPSS) programmed for Windows, version 20.

### Results

All four hundred children were enrolled in this study; 43.8% were male and 56.2% were females with age nominal maximum and minimum of 18 and 0. The average of blood parameters obtained by complete haemogram for the all study group was Hb 11.6 ± 2.4 g/dl, PCV 35.5%, MCV 82.6 fl, MCH 27.1 pg, MCHC 32.7 g, TWBCs 7.9 ± 6.3 × 10^3^/µl, RBCs count 4.5 ± 1.5 × 1012/l and platelets count was 285 ± × 134.6 × 10^3^/µl.

Individuals with SCA (Hb SS) consistently had a low average of blood Hb concentration 7.0 ± 1.1 g/dl, normal MCV 92.4 ± 7.4 fl and high TWBCs 17.3 ± 15.1 × 103/µL in comparison to the SCT. Then individuals with SCT (Hb AS) had haematological parameters similar to normal individuals (Table 1).

**Table 1:** The hematological parameters of study normal group (Hb AA), sickle trait (Hb AS) and sickle cell disease (Hb SS) groups

Blood parameters	AA	AS	SS
RBCs (10^12^/l)	4.44 ± 0.84	4.19 ± 0.68	6.46 ± 6.9
Hb (g/dl)	11.83 ± 2.3	11.28 ± 1.8	7.0 ± 1.1
HCT (%)	36.3 ± 6.5	33.9 ± 5.4	22.3 ± 3.0
MCHC (g/dl)	32.6 ± 1.8	33.3 ± 1.6	31.5 ± 2.6
MCV (fl)	82.5 ± 8.3	80.3 ± 9.3	92.4 ± 7.4
MCH (pg)	27.0 ± 3.1	27.8 ± 2.2	29.1 ± 3.2
TWBCs (10^3^/µl)	7.6 ± 5.5	7.8 ± 4.9	17.3 ± 15.1
PITs (10^3^/µl)	278.1 ± 132.7	298.4 ± 121.0	400.4 ± 173.7

The participants belong to 41 tribes and these represent most of the Northern Darfur tribes, Zaghawa, Fur and Bartey were the predominant tribes (Additional file 2: Table S1).

The prevalence of sickle cell anaemia by Hb electrophoresis among 400 children patients was 59 (14.8%). SCT (Hb AS) and SCD (Hb SS) positive patients were 11.3% and 3.5% respectively. Statistically, significant association (P.value = 0.00) between the sickle cell anaemia and normal blood haemoglobin.

The homozygous form of HbSS was found in five tribes, stratified as follows: Housa 5 (35.7%) followed by Fur 4 (28.6%), Bartey and Zaghawa 2 (14.3%) and finally, Arab-Bashir were 1 (7.1%), (Table 2). While the heterozygous form, (HbAS) was detected in sixteen tribes of Northern Darfur State.

**Table 2:** Frequency of (Hb SS) and (Hb AS) among tribes of study group

Tribes	Frequency (%)	
	SS	AS
Housa	5 (35.7)	2 (4.4)
Fur	4 (28.6)	11 (24.4)
Zagawa	2 (14.3)	10 (22.2)
Bartey	2 (14.3)	5 (11.1)
Arab Basher	1 (7.1)	
Kenen		4 (8.9)
Ziadya		2 (4.4)
Mema		2 (4.4)
Tongour		1 (2.2)
Fallata		1 (2.2)
Etafaat		1 (2.2)
Barno		1 (2.2)
Tama		1 (2.2)
Bin Hussain		1 (2.2)
Mararet		1 (2.2)
Rezegat		1 (2.2)
Awladrashed		1 (2.2)
Total	14 (100)	45 (100)

**Fig. 1:**
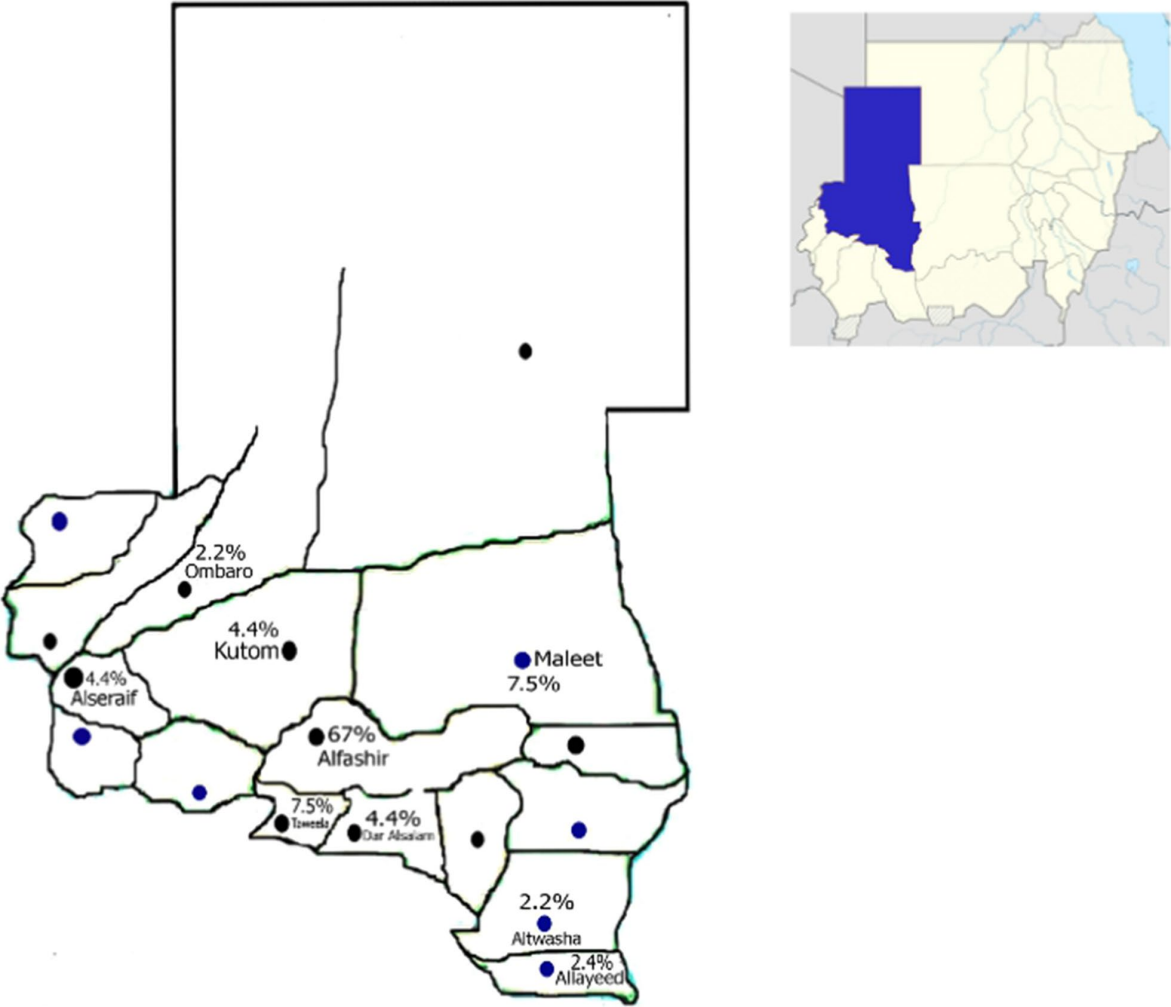
Prevalence of sickle cell trait in 18 localities in Northern Darfur State, Sudan. Prevalence range from 2.2 to 67%. Sincere thanks to the Extended Immunization Program, North Darfur State whom provide these maps for common uses

Sickle cell trait was found in nine localities of 18 localities of Northern Darfur State (Fig. 1). Altwasha and Ombaro regions had the lowest prevalence, being about 2.2%. While Elfashir region had prevalence reach to 67%.

### Discussion

World Health Organization considers sickle cell anaemia as a public health concern Worldwide; it is the most common inherited blood disorder in sub-Saharan Africa [18].

The prevalence of sickle cell anaemia in various regions of Sudan is about 0.8% in central Sudan to 30.4% in Western Sudan as revealed by the available data. The Messeryia tribe in Kordofan and Darfur represent the highest frequency of sickle cell anaemia. High prevalence of sickle cell gene was documented among the population that migrated from Western Africa reported in Al Gadarif state in Eastern Sudan region [11].

In Sudan, epidemiological studies about sickle cell disease are generally scarce. We believe this study represents the first attempt to determine the prevalence of sickle cell anaemia among children in Al Fashir hospital. Here there is no neonatal screening program and a paucity of data regarding children hospitalized by sickle cell anaemia in the North Darfur region.

Admittedly, this study is of children who had some reason to be attending hospital, the prevalence of sickle cell anaemia among 400 children patients was 59 (14.8%); lower than (26.9%)/1000 the prevalence of SCA documented in a recent similar study by Stephen et al. [19] in Nigeria, our sample was smaller than that of Nigeria.

The highest prevalence of sickle cell trait was detected in Elfashir region 67%, compared with low prevalence were detected in other regions of the state. This is referred to the fact that Elfashir is the capital of the state where the populations were accumulated due to the shortage of social services out site big cities and to the presences of displaced camps as a result of civil war.

This study reported (Hb AS) and (Hb SS) positive patients were 11.3% and 3.5% respectively.

Half of 14 (3.5%) of Hb SS patients were known by their parents as SCD patients because they are hospitalized more than a once, while the others were identified as SCD patients for the first time by this study. However, our results agreed with the studies reported in North Darfur, West Kordofan and Heglig area in Western Sudan by Elderderry et al. [20], Daak et al. [13] and Mustafa et al. [21] the prevalence of SCT and SCD were (10.1%) (1.5%), (3.6%) (21.2%), (52%) and (14%) respectively. Similar results were documented in Cameroon, Kenya, and Uganda by Sack et al. [4], Musoriza [22], Ndeezi et al. [23] (16.8%) & (0.75%), (19.0%) & (0.9%), (13.3%) & (0.7%) respectively. The high prevalence of SCA among patients attending Al Fashir hospital refer to the fact that the majority of them from districts rural areas of Darfur region with a low socio-economic background who are furthermore often illiterates.

Our results showed that individuals with SCA (Hb SS) consistently had a low average of Hb 7.0 ± 1 g/dl, normal MCV 92.4 ± 7 fl and high TWBCs 17.3 ± 15 × 103/µl compared to the normal individuals’ group. While individuals with SCT (Hb AS) had haematological parameters similar to the normal individuals, where SCA individuals are not suffering from the blood disorder, and if anaemia is present, it would be because of factors (such as iron deficiency) other than sickling state. The same finding was described by Elderderry et al. [20] in Northern Darfur tribes, Mustafa et al. [21] in Higlig Kordofan and Macharia et al. [5] in Kenya (Hb SS) patients mean Hb g/dl, MCV fl and TWBCs × 103/µl were [(6.95), (92.2) & (18.9)], [6.6), (92.5) & (20.4)] and [(6.31), (80.5) & (21.7)] respectively. The actual anaemia of the SCD is caused by haemolysis, the destruction of the sickle red cells inside the spleen. Although the bone marrow attempts to compensate by creating new red cells, it does not match the rate of destruction, by the other side, the infectious complications often lead to leukocytosis.

In this study SCA (Hb SS) was detected in the Negroid ethnic group; they are a part of Nilo-Saharan language family of North Darfur localities such as Housa 35.7, Fur 28.6%, Zagawa and Bartey 14.3%. While the heterozygous form, (HbAS) was detected in 16 tribes of Northern Darfur State.

The presence of (Hb SS) is already well documented among Kordofan and Darfur region inhabitants, especially among Albaggara, an Afro-Arab constellation of tribes with predominantly African descent, in addition to Bederia, Fulani, Messeryia, Hummer, Berge, Fur and Masaleet [11]. Our findings agreed with (Hb SS & Hb AS) reported by Elderderry et al. [20] among North Darfur tribes were The homozygous form of HbSS was found in four tribes, Housa 10.26%, Fur 3.1%, Bartey 2.7% and Zagawa 1.74%. The heterozygous form (HbAS) was found as 13.2% Fur, 12.8% Housa, 10.5% Ziadya, 9.6% Zagawa 9.5% and Bartey. The high prevalence of SCD and SCT in Northern Darfur tribes is probably due to the high rates of consanguinity, and first cousin marriages there, which could increase the prevalence of autosomal recessive diseases. Moreover, the lack of public health measures and services for the prevention of genetic disorders in general, in addition to the selective termination of pregnancy of an affected fetus is illegal in Sudan.

### Conclusions

This study revealed a high prevalence of sickle cell anaemia among children patients attending Al Fashir hospital, which was not previously established in this hospital.

## Recommendations

Sickle cell disease is a largely neglected leading cause of mortality among children in African countries.

Newborn screening is needed in this region of western Sudan due to the huge immigration of West African tribes carrying high prevalence of SCA, in addition to the illiteracy, consanguineous marriage, closure societies, and lack of medical counselling and prevention from genetic disorders in general.

SCA control programs are needed in the Darfur area to establish premarital screening, health education, and immunization and to collect, analyze of data and publicize the outcome.

Studies to quantify the public health burden of SCA are required to be conducted.

## Limitation

Although the present study is the first to detect the prevalence of sickle cell anaemia among the tribes attending the pediatric clinic in Al Fashir Teaching Hospital during January to February 2018 and not include all the tribes of Northern Darfur populations.

In the present study, haemoglobin electrophoresis was conducted to detect 14 types of haemoglobin, rather than Hb S, no abnormal haemoglobin was detected.

## Supplementary information

**Additional file 1.** Gender, age, tribes, CBC parameters and Hb electrophoresis of the study population.

**Additional file 2.** Examples for the study populations families informed consents.

## Data Availability

The raw dataset analyzed during the current study are presented within an additional Microsoft Word Document file.

## Abbreviations

MCH: mean cell haemoglobin
MCHC: mean cell haemoglobin concentration
MCV: mean cell volume
RBCs: red blood cells
WBC: white blood cells
USA: United States of America
