# Supplementary material for: Prevalence of sickle cell disease and sickle cell trait among children admitted to Al Fashir Teaching Hospital North Darfur State, Sudan
Source: BMC Res Notes. 2019 Oct 16;12:659. doi: 10.1186/s13104-019-4682-5 (PMC6796395; doi:10.1186/s13104-019-4682-5)
Supplement: Supplementary file 1 — Additional file 1. Gender, age, tribes, CBC parameters and Hb electrophoresis of the study population. [file 13104_2019_4682_MOESM1_ESM.docx]

**Prevalence of βs gene**

| **Hb Electro** | **PLTs** | **TWBCs** | **MCHC** | **MCH** | **MCV** | **RBCs** | **PCV** | **Hb** | **Tribe** | **Age/ Years** | **Sex** | **No** |
| --- | --- | --- | --- | --- | --- | --- | --- | --- | --- | --- | --- | --- |
| S | 199 | 10.3 | 30.6 | 21.4 | 70.0 | 4.99 | 35.0 | 10.7 | 13 | Less than 1 | M | 1 |
| S | 347 | 9.3 | 31.2 | 28.8 | 92.2 | 2.19 | 20.2 | 6.3 | 17 | 8-13 | M | 2 |
| S | 200 | 12.7 | 35.6 | 33.2 | 93.2 | 1.90 | 17.7 | 6.6 | 17 | 1-7 | F | 3 |
| S | 190 | 30.6 | 32.5 | 29.6 | 91.1 | 2.91 | 26.5 | 8.6 | 21 | 8-13 | F | 4 |
| S | 189 | 6.7 | 31.2 | 31.4 | 100.5 | 4.08 | 41.0 | 12.8 | 22 | 14-18 | F | 5 |
| S | 493 | 18.4 | 31.5 | 26.9 | 85.4 | 3.16 | 27.0 | 8.5 | 4 | Less than 1 | M | 6 |
| S | 365 | 19.6 | 31.4 | 27.8 | 88.5 | 2.52 | 22.3 | 7.0 | 23 | 8-13 | F | 7 |
| N | 437 | 4.3 | 30.6 | 25.2 | 82.3 | 4.01 | 33.0 | 10.1 | 13 | 1-7 | F | 8 |
| N | 123 | 1.6 | 33.1 | 27.0 | 81.4 | 3.82 | 31.1 | 10.3 | 15 | 1-7 | M | 9 |
| N | 137 | 8.2 | 30.6 | 24.5 | 80.0 | 4.45 | 35.6 | 10.9 | 1 | Less than 1 | M | 10 |
| N | 117 | 18.7 | 27.1 | 24.9 | 92.1 | 3.93 | 36.2 | 9.8 | 1 | 1-7 | M | 11 |
| S | 489 | 10.0 | 32.6 | 23.3 | 71.4 | 4.51 | 32.2 | 10.5 | 13 | Less than 1 | M | 12 |
| N | 211 | 1.2 | 31.7 | 28.1 | 88.8 | 5.62 | 49.9 | 15.8 | 1 | 14-18 | M | 13 |
| N | 269 | 2.1 | 32.4 | 29.3 | 90.7 | 4.60 | 41.7 | 13.5 | 4 | 8-13 | M | 14 |
| N | 320 | 2.1 | 33.3 | 28.5 | 85.4 | 4.32 | 36.9 | 12.3 | 6 | 14-18 | M | 15 |
| S | 227 | 2.8 | 34.6 | 27.8 | 80.3 | 4.49 | 35.8 | 12.4 | 26 | 1-7 | M | 16 |
| N | 413 | 3.1 | 31.0 | 22.2 | 71.8 | 4.54 | 32.6 | 10.1 | 6 | 1-7 | M | 17 |
| N | 99 | 1.0 | 33.1 | 29.7 | 89.6 | 5.39 | 48.3 | 16.0 | 28 | 14-18 | M | 18 |
| S | 494 | 3.9 | 34.6 | 26.4 | 76.1 | 4.40 | 33.5 | 11.6 | 6 | 1-7 | F | 19 |
| N | 350 | 5.4 | 31.5 | 27.6 | 87.5 | 4.39 | 38.4 | 12.1 | 1 | 8-13 | F | 20 |
| N | 225 | 2.9 | 33.3 | 31.0 | 93.0 | 5.16 | 48.0 | 16.0 | 6 | 14-18 | M | 21 |
| N | 278 | 5.4 | 33.5 | 28.3 | 84.4 | 4.95 | 41.8 | 14.0 | 1 | 14-18 | F | 22 |
| N | 219 | 5.5 | 34.0 | 33.3 | 98.1 | 4.23 | 41.5 | 14.1 | 23 | 14-18 | F | 23 |
| N | 300 | 6.3 | 34.0 | 28.3 | 83.2 | 5.06 | 42.1 | 14.3 | 5 | 8-13 | F | 24 |
| N | 266 | 1.6 | 32.4 | 30.1 | 93.0 | 4.28 | 39.8 | 12.9 | 1 | 14-18 | F | 25 |
| N | 211 | 1.2 | 31.7 | 28.1 | 88.8 | 5.62 | 49.9 | 15.8 | 1 | 8-13 | M | 26 |
| N | 205 | 1.8 | 32.4 | 29.1 | 89.8 | 5.32 | 47.8 | 15.5 | 1 | 8-13 | M | 27 |
| N | 310 | 2.1 | 29.1 | 25.0 | 86.0 | 4.56 | 39.2 | 11.4 | 2 | 8-13 | F | 28 |
| N | 295 | 4.2 | 31.7 | 28.1 | 88.6 | 4.84 | 42.9 | 13.6 | 13 | 8-13 | F | 29 |
| N | 219 | 2.9 | 31.9 | 29.8 | 93.4 | 4.83 | 45.1 | 14.4 | 1 | 1-7 | F | 30 |
| N | 272 | 3.3 | 32.6 | 26.7 | 82.0 | 4.72 | 38.7 | 12.6 | 1 | 1-7 | M | 31 |
| N | 162 | 4.8 | 32.5 | 29.0 | 89.4 | 4.82 | 43.1 | 14.0 | 1 | 14-18 | F | 32 |
| N | 156 | 9.4 | 33.1 | 27.9 | 84.2 | 4.56 | 38.4 | 12.7 | 1 | 1-7 | F | 33 |
| N | 197 | 2.7 | 32.7 | 28.5 | 87.1 | 4.95 | 43.1 | 14.1 | 3 | 14-18 | M | 34 |
| N | 277 | 6.1 | 31.9 | 27.6 | 86.6 | 4.63 | 40.1 | 12.8 | 3 | 14-18 | F | 35 |
| N | 261 | 5.1 | 31.8 | 28.8 | 90.4 | 5.32 | 48.1 | 15.3 | 3 | 14-18 | F | 36 |
| N | 580 | 8.4 | 31.5 | 24.6 | 78.2 | 4.35 | 34.0 | 10.7 | 3 | 1-7 | M | 37 |
| S | 352 | 4.8 | 33.1 | 29.2 | 88.1 | 4.87 | 42.9 | 14.2 | 3 | 14-18 | F | 38 |
| S | 313 | 3.6 | 33.1 | 28.9 | 87.5 | 4.56 | 39.9 | 13.2 | 3 | 14-18 | F | 39 |
| N | 313 | 4.3 | 32.7 | 26.9 | 82.3 | 4.57 | 37.6 | 12.3 | 3 | 1-7 | F | 40 |
| N | 122 | 4.6 | 27.9 | 20.1 | 71.9 | 4.13 | 29.7 | 8.3 | 1 | 14-18 | M | 41 |
| SS | 478 | 6.3 | 31.6 | 32.9 | 104.0 | 2.25 | 23.4 | 7.4 | 6 | 8-13 | F | 42 |
| SS | 531 | 11.5 | 28.0 | 28.3 | 101.1 | 2.72 | 27.5 | 7.7 | 6 | 1-7 | F | 43 |
| S | 297 | 11.0 | 32.4 | 28.1 | 86.9 | 5.19 | 45.1 | 14.6 | 1 | 14-18 | F | 44 |
| N | 459 | 10.3 | 31.2 | 27.4 | 87.7 | 4.31 | 37.8 | 11.8 | 3 | 1-7 | F | 45 |

| N | 404 | 5.9 | 33.1 | 28.5 | 86.3 | 4.73 | 40.8 | 13.5 | 1 | 8-13 | M | 46 |
| --- | --- | --- | --- | --- | --- | --- | --- | --- | --- | --- | --- | --- |
| N | 276 | 4.0 | 35.3 | 31.3 | 88.5 | 5.66 | 50.1 | 17.7 | 3 | 14-18 | M | 47 |
| N | 289 | 4.2 | 32.4 | 29.7 | 91.6 | 4.62 | 42.3 | 13.7 | 9 | 14-18 | M | 48 |
| N | 213 | 7.0 | 32.8 | 29.0 | 88.5 | 4.59 | 40.6 | 13.3 | 10 | 14-18 | F | 49 |
| N | 283 | 5.3 | 33.1 | 30.5 | 92.3 | 4.52 | 41.7 | 13.8 | 11 | 8-13 | F | 50 |
| N | 311 | 7.1 | 30.9 | 22.7 | 73.6 | 5.68 | 41.8 | 12.9 | 1 | 14-18 | F | 51 |
| N | 269 | 3.8 | 32.6 | 30.2 | 92.7 | 4.40 | 40.8 | 13.3 | 1 | 14-18 | F | 52 |
| N | 276 | 4,6 | 30.6 | 27.8 | 90.7 | 4.10 | 37.2 | 11.4 | 1 | 8-13 | F | 53 |
| N | 184 | 1.2 | 32.5 | 29.7 | 91.6 | 4.54 | 41.6 | 13.5 | 4 | 14-18 | F | 54 |
| N | 323 | 2.1 | 32.4 | 31.4 | 97.1 | 3.85 | 37.4 | 12.1 | 3 | 14-18 | F | 55 |
| N | 166 | 4.5 | 32.2 | 28.2 | 87.6 | 5.82 | 51.0 | 16.4 | 4 | 14-18 | M | 56 |
| N | 87 | 1.6 | 32.2 | 26.3 | 81.6 | 4.19 | 34.2 | 11.0 | 4 | 14-18 | F | 57 |
| N | 26 | 9.2 | 30.3 | 23.5 | 77.6 | 4.38 | 34.0 | 10.3 | 1 | 1-7 | M | 58 |
| N | 93 | 2.9 | 33.2 | 26.7 | 80.4 | 2.81 | 22.6 | 7.5 | 13 | 8-13 | M | 59 |
| N | 453 | 2.3 | 30.7 | 26.2 | 85.2 | 4.32 | 36.8 | 11.3 | 3 | 1-7 | M | 60 |
| N | 452 | 7.5 | 32.5 | 25.7 | 79.2 | 4.32 | 34.2 | 11.1 | 13 | Less than 1 | M | 61 |
| N | 380 | 2.2 | 31.5 | 25.9 | 82.1 | 4.48 | 36.8 | 11.6 | 1 | 8-13 | F | 62 |
| N | 169 | 10.5 | 31.6 | 26.6 | 84.3 | 3.94 | 33.2 | 10.5 | 1 | 1-7 | F | 63 |
| N | 400 | 8.3 | 31.3 | 25.0 | 80.0 | 4.24 | 33.9 | 10.6 | 13 | 1-7 | F | 64 |
| N | 114 | 5.1 | 33.2 | 29.1 | 87.6 | 4.37 | 38.3 | 12.7 | 1 | 14-18 | F | 65 |
| N | 351 | 3.8 | 32.4 | 31.1 | 95.9 | 4.66 | 44.7 | 14.5 | 18 | 8-13 | F | 66 |
| N | 278 | 4.2 | 32.5 | 32.1 | 98.8 | 4.21 | 41.6 | 13.5 | 8 | 8-13 | F | 67 |
| N | 301 | 5.0 | 31.8 | 27.0 | 84.8 | 4.86 | 41.2 | 13.1 | 1 | 8-13 | F | 68 |
| N | 273 | 6.4 | 33.2 | 31.4 | 94.3 | 4.21 | 39.7 | 13.2 | 16 | 14-18 | F | 69 |
| N | 224 | 3.3 | 32.7 | 31.6 | 96.5 | 4.56 | 44.0 | 14.4 | 19 | 14-18 | F | 70 |
| N | 336 | 5.0 | 33.3 | 31.0 | 92.9 | 4.94 | 45.9 | 15.3 | 1 | 14-18 | F | 71 |
| N | 190 | 2.6 | 32.4 | 28.5 | 87.9 | 4.78 | 42.0 | 13.6 | 20 | 14-18 | F | 72 |
| N | 309 | 5.3 | 33.3 | 28.9 | 86.9 | 5.40 | 46.9 | 15.6 | 5 | 14-18 | F | 73 |
| N | 262 | 13.6 | 34.9 | 29.2 | 83.6 | 5.00 | 41.8 | 15.0 | 12 | 14-18 | F | 74 |
| N | 194 | 4.6 | 34.7 | 26.9 | 77.4 | 4.65 | 36.0 | 14.2 | 6 | 8-13 | M | 75 |
| N | 216 | 5.1 | 32.8 | 31.0 | 94.5 | 4.65 | 46.6 | 13.8 | 6 | 8-13 | M | 76 |
| N | 44 | 4.1 | 32.1 | 32.0 | 99.6 | 2.75 | 27.4 | 13.9 | 25 | 1-7 | M | 77 |
| N | 32 | 7.7 | 32.7 | 26.2 | 80.1 | 4.08 | 32.7 | 12.0 | 13 | 14-18 | M | 78 |
| N | 138 | 7.0 | 27.0 | 18.1 | 67.1 | 4.19 | 28.1 | 12.7 | 9 | 1-7 | F | 79 |
| N | 208 | 7.7 | 33.3 | 29.0 | 85.5 | 5.2 | 44.3 | 15.0 | 4 | 14-18 | F | 80 |
| N | 210 | 5.2 | 33.2 | 26.8 | 80.4 | 4.89 | 39.3 | 13.1 | 1 | 1-7 | M | 81 |
| N | 206 | 4.3 | 32.8 | 28.0 | 88.9 | 4.50 | 39.6 | 13.0 | 27 | 14-18 | F | 82 |
| S | 239 | 3.4 | 33.2 | 26.4 | 79.5 | 4.35 | 34.6 | 11.5 | 1 | 8-13 | F | 83 |
| N | 263 | 9.8 | 32.2 | 26.7 | 82.9 | 4.91 | 40.7 | 13.1 | 23 | 14-18 | M | 84 |
| S | 432 | 6.7 | 33.4 | 26.7 | 80.0 | 4.45 | 35.6 | 11.9 | 1 | 14-18 | M | 85 |
| N | 423 | 7.9 | 29.9 | 20.6 | 68.8 | 5.0 | 34.4 | 10.3 | 3 | 1-7 | M | 86 |

| N | 211 | 7.2 | 32.5 | 27.8 | 85.5 | 4.28 | 36.6 | 11.9 | 3 | 8-13 | F | 87 |
| --- | --- | --- | --- | --- | --- | --- | --- | --- | --- | --- | --- | --- |
| N | 270 | 10.5 | 34.2 | 25.2 | 73.7 | 4.41 | 32.5 | 11.1 | 16 | Less than 1 | M | 88 |
| N | 331 | 10.3 | 33.5 | 24.9 | 74.2 | 4.34 | 32.2 | 10.8 | 1 | 1-7 | M | 89 |
| N | 167 | 12.0 | 34.3 | 26.3 | 74.6 | 4.52 | 33.7 | 11.9 | 1 | Less than 1 | F | 90 |
| N | 494 | 11.8 | 32.8 | 24.6 | 74.9 | 4.23 | 31.7 | 10.4 | 3 | Less than 1 | F | 91 |
| N | 138 | 12.0 | 34.5 | 28.7 | 83.3 | 3.83 | 31.9 | 11.0 | 21 | 8-13 | M | 92 |
| N | 346 | 7.3 | 33.5 | 24.6 | 73.3 | 4.23 | 31.0 | 10.4 | 1 | 1-7 | F | 93 |
| N | 253 | 10.3 | 32.8 | 23.0 | 70.0 | 4.27 | 29.9 | 9.8 | 1 | Less than 1 | M | 94 |
| N | 453 | 14.5 | 33.4 | 27.2 | 81.3 | 4.49 | 36.5 | 12.2 | 15 | Less than 1 | F | 95 |
| S | 337 | 6.7 | 34.2 | 28.1 | 82.1 | 4.02 | 33.0 | 11.3 | 3 | Less than 1 | F | 96 |
| N | 418 | 6.4 | 36.5 | 27.6 | 75.7 | 4.20 | 31.8 | 11.6 | 1 | 1-7 | F | 97 |
| N | 480 | 11.8 | 28.9 | 22.6 | 78.1 | 4.07 | 31.8 | 9.2 | 1 | Less than 1 | M | 98 |
| N | 195 | 13.3 | 34.7 | 28.4 | 81.9 | 2.15 | 17.6 | 6.1 | 1 | Less than 1 | F | 99 |
| N | 560 | 8.1 | 33.4 | 29.6 | 88.5 | 3.65 | 32.3 | 10.8 | 15 | Less than 1 | M | 100 |
| SS | 174 | 8.1 | 35.8 | 30.4 | 84.8 | 2.70 | 22.9 | 8.2 | 13 | 1-7 | M | 101 |
| S | 365 | 9.0 | 32.9 | 23.8 | 72.5 | 4.11 | 29.8 | 9.8 | 3 | Less than 1 | F | 102 |
| N | 328 | 7.5 | 33.4 | 25.6 | 76.6 | 4.10 | 31.4 | 10.5 | 15 | 1-7 | M | 103 |
| N | 445 | 6.1 | 31.5 | 21.1 | 66.9 | 4.17 | 27.9 | 8.8 | 13 | Less than 1 | M | 104 |
| N | 500 | 8.1 | 33.1 | 24.3 | 73.3 | 4.49 | 32.9 | 10.9 | 1 | 1-7 | M | 105 |
| S | 150 | 8.2 | 34.1 | 28.8 | 84.3 | 4.00 | 33.7 | 11.5 | 3 | 1-7 | M | 106 |
| N | 284 | 8.5 | 34.0 | 23.8 | 70.0 | 4.07 | 28.5 | 9.7 | 15 | Less than 1 | M | 107 |
| N | 226 | 15.2 | 35.0 | 29.0 | 82.9 | 1.93 | 16.0 | 5.6 | 1 | 1-7 | M | 108 |

| S | 650 | 7.8 | 33.3 | 26.3 | 78.8 | 4.53 | 35.7 | 11.9 | 27 | 1-7 | F | 109 |
| --- | --- | --- | --- | --- | --- | --- | --- | --- | --- | --- | --- | --- |
| N | 88 | 4.7 | 34.5 | 25.5 | 73.7 | 3.73 | 27.5 | 9.5 | 15 | 1-7 | M | 110 |
| N | 452 | 8.0 | 31.2 | 24.4 | 37.8 | 4.84 | 37.8 | 11.8 | 1 | 1-7 | M | 111 |
| S | 232 | 5.0 | 32.6 | 25.8 | 79.1 | 4.46 | 35.3 | 11.5 | 3 | 1-7 | F | 112 |
| N | 63 | 5.9 | 33.2 | 29.4 | 88.3 | 3.85 | 34.0 | 11.3 | 33 | 8-13 | F | 113 |
| N | 223 | 7.5 | 32.7 | 26.7 | 81.7 | 5.69 | 46.5 | 15.2 | 1 | 8-13 | F | 114 |
| S | 196 | 6.9 | 33.8 | 27.3 | 34.0 | 4.21 | 34.0 | 11.5 | 1 | 1-7 | M | 115 |
| S | 195 | 5.3 | 34.4 | 27.9 | 81.1 | 4.34 | 35.2 | 12.1 | 15 | Less than 1 | M | 116 |
| N | 65 | 3.1 | 34.1 | 28.2 | 82.8 | 4.18 | 34.6 | 11.8 | 16 | 8-13 | F | 117 |
| N | 250 | 14.6 | 33.6 | 25.8 | 76.7 | 4.34 | 33.3 | 11.2 | 3 | Less than 1 | M | 118 |
| N | 70 | 15.1 | 33.3 | 26.4 | 79.3 | 3.52 | 27.9 | 9.3 | 33 | 1-7 | M | 119 |
| S | 126 | 7.5 | 33.1 | 25.4 | 76.5 | 4.26 | 32.6 | 10.8 | 13 | Less than 1 | F | 120 |
| N | 373 | 14.4 | 32.7 | 24.3 | 74.3 | 3.46 | 25.7 | 8.4 | 13 | Less than 1 | F | 121 |
| N | 780 | 13.4 | 27.9 | 16.7 | 60.0 | 5.32 | 31.9 | 8.9 | 13 | 1-7 | M | 122 |
| N | 100 | 11.2 | 32.6 | 22.9 | 70.1 | 3.85 | 27.0 | 8.8 | 1 | 1-7 | F | 123 |
| S | 306 | 4.2 | 31.8 | 26.1 | 82.1 | 4.59 | 37.7 | 12.0 | 29 | Less than 1 | M | 124 |
| N | 435 | 10.6 | 31.1 | 22.4 | 72.0 | 5.00 | 36.0 | 11.2 | 3 | Less than 1 | M | 125 |
| N | 294 | 4.2 | 32.3 | 26.2 | 81.2 | 4.58 | 37.2 | 12.0 | 3 | Less than 1 | F | 126 |
| N | 563 | 27.3 | 33.5 | 30.4 | 90.8 | 3.06 | 27.8 | 9.3 | 16 | Less than 1 | F | 127 |
| N | 198 | 3.8 | 34.3 | 30.2 | 88.0 | 3.08 | 27.1 | 9.3 | 3 | 1-7 | F | 128 |
| N | 63 | 14.0 | 31.6 | 23.3 | 73.8 | 3.47 | 25.6 | 8.1 | 1 | 1-7 | M | 129 |
| N | 369 | 3.7 | 32.6 | 26.5 | 81.4 | 4.52 | 36.8 | 12.0 | 33 | 11 Y | M | 130 |
| S | 251 | 3.6 | 31.8 | 26.2 | 82.4 | 4.54 | 37.4 | 11.9 | 1 | 1-7 | M | 131 |
| N | 120 | 16.6 | 25.8 | 20.4 | 79.0 | 3.09 | 24.4 | 6.3 | 13 | 1-7 | F | 132 |

| S | 192 | 6.6 | 32.9 | 23.9 | 72.6 | 3.98 | 28.9 | 9.5 | 15 | Less than 1 | F | 133 |
| --- | --- | --- | --- | --- | --- | --- | --- | --- | --- | --- | --- | --- |
| SS | 88 | 7.5 | 31.0 | 27.9 | 89.9 | 2.87 | 25.8 | 8.0 | 1 | 14-18 | F | 134 |
| N | 245 | 2.9 | 35.5 | 27.2 | 76.6 | 3.20 | 24.5 | 8.7 | 16 | 1-7 | M | 135 |
| N | 278 | 11.1 | 31.9 | 20.9 | 65.4 | 4.89 | 32.0 | 10.2 | 15 | 1-7 | M | 136 |
| N | 287 | 10.4 | 30.4 | 20.7 | 68.2 | 4.44 | 30.3 | 9.2 | 11 | 1-7 | M | 137 |
| S | 329 | 8.8 | 33.8 | 25.1 | 74.4 | 3.94 | 29.3 | 9.9 | 13 | 1-7 | F | 138 |
| N | 189 | 1.4 | 32.5 | 24.6 | 75.7 | 3.37 | 25.5 | 8.3 | 3 | 8-13 | M | 139 |
| N | 482 | 8.1 | 30.7 | 20.5 | 66.8 | 4.43 | 29.6 | 9.1 | 33 | Less than 1 | M | 140 |
| N | 213 | 5.3 | 31.6 | 29.8 | 94.2 | 3.12 | 29.4 | 9.3 | 15 | 1-7 | M | 141 |
| N | 406 | 7.1 | 33.7 | 26.9 | 79.7 | 4.69 | 37.4 | 12.6 | 33 | 1-7 | F | 142 |
| S | 269 | 6.8 | 34.9 | 28.0 | 80.1 | 4.22 | 33.8 | 11.8 | 13 | Less than 1 | M | 143 |
| S | 313 | 5.6 | 33.6 | 25.7 | 76.7 | 3.69 | 28.3 | 9.5 | 1 | 1-7 | F | 144 |
| N | 134 | 6.9 | 33.4 | 26.0 | 77.7 | 4.35 | 33.8 | 11.3 | 4 | 1-7 | F | 145 |
| S | 296 | 4.6 | 33.1 | 26.3 | 79.6 | 4.52 | 36.0 | 11.9 | 21 | 1-7 | F | 146 |
| S | 293 | 4.5 | 33.0 | 26.4 | 80.2 | 4.50 | 36.1 | 11.9 | 21 | Less than 1 | F | 147 |
| S | 290 | 4.8 | 32.8 | 26.2 | 79.8 | 4.55 | 36.3 | 11.9 | 3 | 1-7 | M | 148 |
| S | 279 | 4.5 | 33.1 | 26.5 | 80.0 | 4.49 | 35.9 | 11.9 | 1 | 8-13 | M | 149 |
| S | 285 | 5.4 | 33.2 | 27.2 | 81.7 | 4.49 | 36.7 | 12.2 | 3 | 8-13 | F | 150 |
| N | 298 | 7.5 | 32.9 | 26.2 | 79.7 | 4.77 | 38.0 | 12.5 | 16 | 1-7 | M | 151 |
| S | 67 | 5.6 | 39.4 | 31.5 | 79.8 | 3.97 | 31.7 | 12.5 | 1 | 1-7 | M | 152 |
| S | 376 | 5.3 | 34.6 | 25.5 | 73.7 | 4.67 | 34.4 | 11.9 | 1 | 1-7 | M | 153 |
| S | 608 | 8.8 | 32.8 | 26.1 | 79.6 | 4.21 | 33.5 | 11.0 | 16 | 8-13 | F | 154 |
| N | 59 | 4.2 | 34.9 | 28.4 | 81.3 | 4.55 | 37.0 | 12.9 | 1 | 8-13 | M | 155 |
| N | 51 | 5.0 | 37.6 | 30.1 | 80.1 | 4.12 | 33.0 | 12.4 | 3 | 8-13 | M | 156 |
| N | 112 | 5.2 | 34.0 | 27.4 | 80.5 | 4.16 | 33.5 | 11.4 | 1 | 8-13 | F | 157 |
| SS | 137 | 14.0 | 29.4 | 26.4 | 89.9 | 1.78 | 16.0 | 4.7 | 35 | 1-7 | F | 158 |
| N | 389 | 10.8 | 31.7 | 24.1 | 75.9 | 3.82 | 29.0 | 9.2 | 15 | Less than 1 | M | 159 |
| N | 603 | 17.4 | 30.2 | 19.9 | 66.1 | 5.72 | 37.8 | 11.4 | 3 | Less than 1 | F | 160 |
| N | 385 | 6.2 | 31.5 | 21.3 | 67.7 | 5.02 | 34.0 | 10.7 | 4 | 1-7 | F | 161 |
| N | 604 | 10.9 | 30.8 | 20.5 | 66.6 | 4.88 | 32.5 | 10.0 | 4 | Less than 1 | F | 162 |
| N | 589 | 6.9 | 29.9 | 23.9 | 79.8 | 4.31 | 34.4 | 10.3 | 3 | Less than 1 | F | 163 |
| N | 348 | 7.3 | 32.5 | 25.9 | 79.6 | 4.06 | 32.3 | 10.5 | 1 | Less than 1 | F | 164 |
| N | 338 | 14.0 | 33.9 | 27.7 | 81.6 | 4.19 | 34.2 | 11.6 | 13 | Less than 1 | F | 165 |
| N | 535 | 11.3 | 31.5 | 23.1 | 73.3 | 4.64 | 34.0 | 10.7 | 16 | 1-7 | F | 166 |
| N | 602 | 17.0 | 32.3 | 26.1 | 80.7 | 4.41 | 35.6 | 11.5 | 1 | Less than 1 | F | 167 |
| N | 585 | 7.3 | 31.5 | 22.2 | 70.4 | 4.50 | 31.7 | 10.0 | 15 | 1-7 | M | 168 |
| S | 347 | 7.2 | 34.0 | 26.9 | 79.0 | 4.43 | 35.0 | 11.9 | 3 | 1-7 | M | 169 |
| N | 372 | 11.6 | 35.2 | 29.0 | 83.3 | 3.28 | 27.0 | 9.5 | 21 | Less than 1 | M | 170 |
| S | 225 | 7.9 | 32.7 | 24.7 | 75.7 | 4.57 | 34.6 | 11.3 | 1 | Less than 1 | M | 171 |
| N | 222 | 7.5 | 33.3 | 26.9 | 80.8 | 2.19 | 17.7 | 5.9 | 1 | 1-7 | M | 172 |
| N | 130 | 8.2 | 34.8 | 27.1 | 77.9 | 3.76 | 29.3 | 10.2 | 16 | 1-7 | M | 173 |
| N | 187 | 7.3 | 36.4 | 30.1 | 82.6 | 3.86 | 31.9 | 11.6 | 21 | 1-7 | F | 174 |
| N | 136 | 6.6 | 33.7 | 24.8 | 73.6 | 4.96 | 36.5 | 12.3 | 21 | 1-7 | M | 175 |
| N | 112 | 7.1 | 34.7 | 31.3 | 90.2 | 3.77 | 34.0 | 11.8 | 21 | 8-13 | F | 176 |
| N | 370 | 8.8 | 33.8 | 25.1 | 74.2 | 3.91 | 29.0 | 9.8 | 4 | Less than 1 | F | 177 |
| N | 411 | 12.7 | 34.5 | 26.1 | 75.8 | 4.17 | 31.6 | 10.9 | 1 | Less than 1 | M | 178 |
| N | 157 | 11.2 | 31.5 | 23.5 | 74.6 | 4.68 | 34.9 | 11.0 | 1 | 1-7 | F | 179 |
| N | 164 | 8.6 | 31.5 | 26.2 | 83.2 | 4.93 | 41.0 | 12.9 | 4 | More than 18 | F | 180 |

| N | 349 | 4.3 | 32.3 | 28.0 | 86.7 | 4.50 | 39.0 | 12.6 | 3 | 14-18 | F | 181 |
| --- | --- | --- | --- | --- | --- | --- | --- | --- | --- | --- | --- | --- |
| N | 286 | 10.2 | 31.8 | 32.9 | 103.5 | 3.46 | 35.8 | 11.4 | 3 | 14-18 | F | 182 |
| S | 230 | 7.2 | 32.3 | 28.0 | 86.5 | 4.65 | 40.2 | 13.0 | 3 | 8-13 | F | 183 |
| N | 238 | 7.7 | 31.1 | 28.3 | 91.0 | 4.21 | 38.3 | 11.9 | 3 | 8-13 | F | 184 |
| N | 201 | 6.7 | 31.1 | 28.2 | 90.4 | 3.87 | 35.0 | 10.9 | 24 | 8-13 | F | 185 |
| N | 370 | 7.7 | 33.4 | 30.7 | 91.7 | 3.49 | 32.0 | 10.7 | 5 | 14-18 | F | 186 |
| N | 314 | 4.6 | 32.3 | 29.8 | 92.4 | 3.28 | 35.3 | 11.4 | 3 | 14-18 | F | 187 |
| N | 186 | 7.1 | 33.9 | 28.9 | 85.3 | 4.50 | 38.4 | 13.0 | 3 | 14-18 | F | 188 |

| N | 286 | 5.0 | 31.3 | 27.8 | 88.7 | 5.15 | 45.7 | 14.3 | 4 | 14-18 | F | 189 |
| --- | --- | --- | --- | --- | --- | --- | --- | --- | --- | --- | --- | --- |
| N | 294 | 3.5 | 32.7 | 27.5 | 84.0 | 4.51 | 37.9 | 12.4 | 1 | 8-13 | F | 190 |
| N | 184 | 4.4 | 34.4 | 29.8 | 86.5 | 4.23 | 36.6 | 12.6 | 3 | 8-13 | F | 191 |
| N | 472 | 5.5 | 29.1 | 23.3 | 80.0 | 4.60 | 36.8 | 10.7 | 13 | 14-18 | F | 192 |
| N | 187 | 13.5 | 32.6 | 28.7 | 87.9 | 4.22 | 37.1 | 12.1 | 13 | 8-13 | F | 193 |
| S | 176 | 5.9 | 33.8 | 29.5 | 87.4 | 4.51 | 39.4 | 13.3 | 3 | 14-18 | F | 194 |
| N | 271 | 10.1 | 31.5 | 26.3 | 83.4 | 4.41 | 36.8 | 11.6 | 3 | 8-13 | F | 195 |
| N | 103 | 5.1 | 32.1 | 26.3 | 82.1 | 4.18 | 34.3 | 11.0 | 3 | 1-7 | F | 196 |
| N | 97 | 4.3 | 31.1 | 27.3 | 87.8 | 4.50 | 39.5 | 12.3 | 3 | 14-18 | F | 197 |
| N | 228 | 5.7 | 32.1 | 27.3 | 85.2 | 4.79 | 40.8 | 13.1 | 4 | 8-13 | F | 198 |

| N | 411 | 8.6 | 30.7 | 24.3 | 79.1` | 5.07 | 40.1 | 12.3 | 4 | 8-13 | M | 199 |
| --- | --- | --- | --- | --- | --- | --- | --- | --- | --- | --- | --- | --- |
| S | 188 | 4.8 | 37.5 | 29.3 | 78.3 | 3.68 | 28.8 | 10.8 | 34 | 1-7 | M | 200 |
| N | 52 | 6.9 | 33.3 | 25.5 | 76.5 | 3.57 | 27.3 | 9.1 | 1 | 1-7 | M | 201 |
| N | 508 | 13.7 | 32.4 | 27.2 | 84.1 | 3.89 | 32.7 | 10.6 | 1 | 1-7 | F | 202 |
| N | 591 | 21.3 | 32.4 | 25.4 | 78.3 | 3.82 | 29.9 | 9.7 | 21 | Less than 1 | F | 203 |
| N | 457 | 16.0 | 32.9 | 22.2 | 67.5 | 4.59 | 31.0 | 10.2 | 1 | Less than 1 | M | 204 |
| N | 624 | 12.9 | 31.7 | 26.3 | 82.8 | 4.00 | 33.1 | 10.5 | 9 | 1-7 | M | 205 |
| N | 128 | 15.0 | 30.0 | 20.0 | 66.7 | 4.54 | 30.3 | 9.1 | 46 | 1-7 | F | 206 |
| N | 414 | 7.8 | 29.5 | 19.8 | 67.1 | 4.59 | 30.8 | 9.1 | 40 | 1-7 | F | 207 |
| N | 549 | 10.8 | 30.4 | 23.5 | 77.3 | 3.83 | 29.6 | 9.0 | 1 | Less than 1 | M | 208 |
| N | 307 | 4.7 | 30.5 | 23.9 | 78.4 | 4.81 | 37.7 | 11.5 | 3 | 1-7 | M | 209 |
| SS | 248 | 35.8 | 30.2 | 25.3 | 83.8 | 2.29 | 19.2 | 5.8 | 13 | 1-7 | F | 210 |
| N | 198 | 12.2 | 31.5 | 27.0 | 85.7 | 1.89 | 16.2 | 5.1 | 15 | Less than 1 | M | 211 |
| N | 165 | 12.6 | 34.0 | 25.7 | 75.5 | 4.21 | 31.8 | 10.8 | 4 | 1-7 | M | 212 |
| N | 51 | 9.4 | 35.2 | 30.0 | 85.2 | 3.57 | 30.4 | 10.7 | 1 | 1-7 | F | 213 |

| N | 56 | 6.0 | 35.0 | 27.7 | 79.2 | 2.31 | 18.3 | 6.4 | 1 | 1-7 | M | 214 |
| --- | --- | --- | --- | --- | --- | --- | --- | --- | --- | --- | --- | --- |
| N | 245 | 21.8 | 33.7 | 30.8 | 91.4 | 3.70 | 33.8 | 11.4 | 21 | 8-13 | F | 215 |
| N | 151 | 2.3 | 31.9 | 25.6 | 80.2 | 3.83 | 30.7 | 9.8 | 1 | 8-13 | M | 216 |
| N | 146 | 2.4 | 32.0 | 25.5 | 79.7 | 3.84 | 30.6 | 9.8 | 16 | Less than 1 | F | 217 |
| N | 273 | 41.7 | 26.5 | 28.5 | 107.7 | 3.79 | 40.8 | 10.8 | 15 | Less than 1 | M | 218 |
| N | 21 | 2.4 | 31.7 | 27.7 | 87.4 | 4.99 | 43.6 | 13.8 | 17 | 8-13 | M | 219 |
| N | 274 | 7.7 | 34.4 | 26.4 | 76.7 | 4.43 | 34.0 | 11.7 | 3 | Less than 1 | M | 220 |
| N | 746 | 15.5 | 29.7 | 18.9 | 63.8 | 5.33 | 34.0 | 10.1 | 13 | 1-7 | M | 221 |
| N | 424 | 5.9 | 33.9 | 24.5 | 72.3 | 5.19 | 37.5 | 12.7 | 15 | 1-7 | F | 222 |
| N | 642 | 9.0 | 33.1 | 25.9 | 78.1 | 3.79 | 29.6 | 9.8 | 1 | Less than 1 | F | 223 |
| N | 479 | 7.3 | 34.5 | 25.9 | 75.1 | 4.25 | 31.9 | 11.0 | 13 | Less than 1 | M | 224 |
| N | 405 | 10.1 | 35.9 | 26.4 | 73.6 | 3.56 | 26.2 | 9.4 | 13 | Less than 1 | M | 225 |
| N | 441 | 11.7 | 30.4 | 29.2 | 95.8 | 1.20 | 11.5 | 3.5 | 21 | 1-7 | F | 226 |
| N | 132 | 5.3 | 35.0 | 26.3 | 75.1 | 4.30 | 32.3 | 11.3 | 1 | 1-7 | M | 227 |
| N | 474 | 5.4 | 34.5 | 28.9 | 83.5 | 3.95 | 33.0 | 11.4 | 58 | 8-13 | F | 228 |

| N | 146 | 5.6 | 30.9 | 25.3 | 82.0 | 4.66 | 38.2 | 11.8 | 48 | 14-18 | F | 229 |
| --- | --- | --- | --- | --- | --- | --- | --- | --- | --- | --- | --- | --- |
| N | 308 | 8.2 | 32.5 | 29.0 | 89.2 | 4.24 | 37.8 | 12.3 | 23 | 8-13 | F | 230 |
| N | 233 | 10.2 | 32.3 | 28.4 | 87.8 | 3.77 | 33.1 | 10.7 | 4 | 8-13 | F | 231 |
| N | 191 | 6.3 | 30.7 | 27.4 | 89.1 | 3.58 | 31.9 | 9.8 | 13 | 1-7 | F | 232 |
| N | 202 | 4.8 | 31.4 | 27.3 | 86.8 | 3.41 | 29.6 | 9.3 | 9 | 8-13 | F | 233 |

| N | 278 | 32.8 | 40.2 | 39.5 | 98.2 | 1.14 | 11.2 | 4.5 | 18 | 8-13 | F | 234 |
| --- | --- | --- | --- | --- | --- | --- | --- | --- | --- | --- | --- | --- |
| N | 283 | 10.6 | 32.5 | 26.6 | 81.8 | 4.33 | 35.4 | 11.5 | 4 | 1-7 | F | 235 |
| N | 366 | 7.2 | 33.2 | 25.7 | 77.2 | 4.95 | 38.2 | 12.7 | 25 | 8-13 | M | 236 |
| N | 324 | 5.6 | 36.3 | 29.9 | 82.3 | 4.12 | 33.9 | 12.3 | 5 | 14-18 | F | 237 |
| N | 79 | 8.5 | 33.9 | 25.0 | 73.8 | 5.00 | 36.9 | 12.5 | 13 | 1-7 | M | 238 |
| N | 291 | 4.6 | 32.7 | 27.5 | 84.1 | 4.54 | 38.2 | 12.5 | 45 | 8-13 | F | 239 |

| N | 288 | 4.4 | 33.6 | 24.7 | 73.5 | 5.10 | 37.5 | 12.6 | 13 | 8-13 | M | 240 |
| --- | --- | --- | --- | --- | --- | --- | --- | --- | --- | --- | --- | --- |
| N | 277 | 5.4 | 34.5 | 26.9 | 77.9 | 4.76 | 37.1 | 12.8 | 13 | 1-7 | F | 241 |
| N | 284 | 6.5 | 33.1 | 25.3 | 76.5 | 4.86 | 37.2 | 12.3 | 6 | 1-7 | F | 242 |
| N | 303 | 4.5 | 33.5 | 28.8 | 86.0 | 4.72 | 40.6 | 13.6 | 6 | 14-18 | M | 243 |
| N | 300 | 4.4 | 34.1 | 29.1 | 85.4 | 4.53 | 38.7 | 13.2 | 6 | 8-13 | F | 245 |
| N | 470 | 11.0 | 32.9 | 28.8 | 87.4 | 4.52 | 39.5 | 13.0 | 6 | 8-13 | M | 246 |
| N | 268 | 6.8 | 34.3 | 30.2 | 88.0 | 4.24 | 37.3 | 12.8 | 45 | 14-18 | F | 247 |
| N | 278 | 6.0 | 34.1 | 26.3 | 77.1 | 4.49 | 34.6 | 11.8 | 13 | 8-13 | M | 248 |

| N | 328 | 10.6 | 35.0 | 26.7 | 76.5 | 10.6 | 30.9 | 10.8 | 1 | 1-7 | F | 249 |
| --- | --- | --- | --- | --- | --- | --- | --- | --- | --- | --- | --- | --- |
| N | 312 | 5.9 | 31.4 | 27.2 | 86.6 | 5.9 | 38.2 | 12.0 | 25 | 14-18 | F | 250 |
| N | 431 | 9.4 | 26.4 | 15.9 | 60.1 | 9.4 | 31.8 | 8.4 | 59 | 1-7 | M | 251 |
| SS | 443 | 13.1 | 31.1 | 28.9 | 92.9 | 13.1 | 23.5 | 7.3 | 6 | Less than 1 | F | 252 |
| SS | 402 | 22.1 | 33.3 | 28.2 | 84.7 | 22.1 | 22.2 | 7.4 | 1 | 1-7 | F | 253 |
| N | 330 | 5.0 | 33.6 | 29.0 | 86.4 | 5.0 | 40.8 | 13.7 | 23 | 14-18 | F | 254 |
| SS | 664 | 16.8 | 32.8 | 32.0 | 97.6 | 16.8 | 20.1 | 6.6 | 3 | 1-7 | M | 254 |
| SS | 596 | 14.3 | 34.7 | 31.4 | 90.4 | 14.3 | 23.6 | 8.2 | 3 | 8-13 | F | 255 |
| SS | 469 | 61.7 | 31.5 | 33.5 | 106.4 | 1.73 | 18.4 | 5.8 | 3 | 1-7 | M | 256 |
| N | 323 | 8.3 | 33.6 | 28.5 | 84.8 | 4.88 | 41.4 | 13.9 | 12 | 8-13 | F | 257 |
| N | 314 | 6.8 | 33.2 | 28.9 | 86.8 | 4.33 | 37.6 | 12.5 | 16 | 1-7 | M | 258 |
| N | 465 | 7.9 | 33.4 | 28.4 | 84.9 | 4.30 | 36.5 | 12.2 | 16 | 1-7 | M | 259 |
| N | 518 | 6.6 | 36.1 | 31.8 | 88.1 | 4.03 | 35.5 | 12.8 | 16 | 8-13 | M | 260 |
| N | 597 | 12.5 | 31.0 | 20.7 | 66.7 | 5.52 | 36.8 | 11.4 | 12 | 1-7 | M | 261 |
| N | 265 | 7.4 | 31.0 | 19.9 | 64.1 | 5.79 | 37.1 | 11.5 | 12 | 8-13 | M | 262 |
| N | 324 | 4.9 | 33.4 | 26 4 | 79.0 | 5.00 | 39.5 | 13.2 | 6 | 1-7 | M | 263 |
| SS | 448 | 15.8 | 26.0 | 21.8 | 84.0 | 2.75 | 23.1 | 6.0 | 3 | Less than 1 | M | 264 |
| N | 286 | 8.1 | 32.5 | 26.5 | 81.7 | 4.75 | 38.8 | 12.6 | 51 | 1-7 | F | 265 |
| N | 236 | 4.1 | 32.7 | 28.5 | 87.0 | 3.62 | 31.5 | 10.3 | 51 | 14-18 | F | 266 |
| S | 209 | 13.6 | 34.9 | 26.7 | 76.5 | 4.68 | 35.8 | 12.5 | 21 | Less than 1 | F | 267 |
| N | 332 | 10.1 | 32.9 | 27.5 | 83.6 | 4.69 | 39.2 | 12.9 | 51 | 8-13 | M | 268 |
| N | 197 | 5.1 | 32.6 | 27.3 | 83.7 | 3.44 | 28.8 | 9.4 | 51 | 8-13 | M | 269 |
| N | 277 | 5.0 | 34.6 | 33.3 | 96.1 | 4.87 | 46.8 | 16.2 | 24 | 14-18 | F | 270 |
| N | 276 | 6.1 | 33.8 | 29.3 | 86.6 | 4.54 | 39.3 | 13.3 | 24 | 14-18 | F | 271 |
| N | 294 | 6.9 | 33.5 | 29.9 | 89.3 | 5.22 | 46.6 | 15.6 | 12 | 14-18 | F | 272 |
| N | 311 | 6.1 | 33.6 | 27.3 | 81.2 | 5.06 | 41.1 | 13.8 | 24 | 1-7 | M | 273 |
| N | 282 | 4.0 | 33.2 | 29.9 | 89.9 | 4.35 | 39.1 | 13.0 | 24 | 8-13 | M | 274 |
| N | 351 | 7.2 | 32.7 | 24.1 | 73.8 | 4.31 | 31.8 | 10.4 | 24 | 1-7 | F | 275 |
| S | 429 | 7.7 | 33.9 | 28.7 | 84.6 | 4.49 | 38.0 | 12.9 | 24 | 1-7 | F | 276 |
| N | 361 | 5.6 | 34.2 | 29.2 | 85.6 | 4.65 | 39.8 | 13.6 | 24 | 8-13 | F | 277 |
| N | 184 | 5.2 | 32.4 | 29.4 | 90.7 | 4.93 | 44.7 | 14.5 | 2 | 8-13 | F | 278 |
| N | 242 | 5.8 | 32.1 | 29.8 | 92.9 | 4.50 | 41.8 | 13.4 | 49 | 14-18 | F | 279 |
| N | 247 | 3.1 | 32.7 | 31.7 | 96.8 | 4.07 | 39.4 | 12.9 | 40 | 8-13 | F | 280 |
| N | 351 | 3.2 | 33.3 | 31.1 | 93.5 | 4.47 | 41.8 | 13.9 | 40 | 14-18 | F | 281 |
| S | 392 | 7.1 | 30.6 | 25.1 | 81.9 | 4.54 | 37.2 | 11.4 | 6 | 14-18 | F | 282 |

| N | 238 | 3.2 | 32.3 | 28.0 | 86.6 | 4.93 | 42.7 | 13.8 | 5 | 14-18 | F | 283 |
| --- | --- | --- | --- | --- | --- | --- | --- | --- | --- | --- | --- | --- |
| N | 300 | 5.2 | 33.1 | 30.0 | 90.6 | 4.90 | 44.4 | 14.7 | 5 | 14-18 | F | 284 |
| N | 194 | 1.4 | 34.4 | 31.5 | 91.7 | 5.17 | 47.4 | 16.3 | 29 | 14-18 | M | 285 |
| N | 300 | 1.9 | 29.2 | 26.2 | 90.0 | 4.61 | 41.5 | 12.1 | 2 | 14-18 | F | 286 |
| N | 270 | 5.0 | 31.4 | 29.3 | 93.3 | 4.81 | 44.9 | 14.1 | 13 | 8-13 | F | 287 |
| N | 271 | 8.0 | 29.8 | 28.0 | 93.7 | 4.47 | 41.9 | 12.5 | 2 | 8-13 | F | 288 |
| N | 168 | 10.5 | 33.0 | 30.2 | 91.4 | 4.87 | 44.5 | 14.7 | 50 | 14-18 | F | 289 |
| N | 201 | 5.6 | 34.4 | 30.1 | 87.4 | 3.96 | 34.6 | 11.9 | 5 | 8-13 | F | 290 |
| N | 399 | 4.8 | 33.5 | 28.9 | 86.2 | 4.64 | 40.0 | 13.4 | 53 | 8-13 | F | 291 |
| N | 445 | 8.0 | 33.0 | 27.0 | 82.1 | 4.29 | 35.2 | 11.6 | 53 | 1-7 | F | 292 |
| N | 305 | 4.4 | 34.4 | 26.9 | 78.3 | 4.98 | 39.0 | 13.4 | 53 | 8-13 | M | 293 |
| N | 274 | 4.0 | 35.1 | 28.3 | 80.8 | 5.01 | 40.5 | 14.2 | 53 | 14-18 | M | 294 |
| N | 248 | 7.2 | 34.1 | 27.4 | 80.2 | 4.64 | 37.2 | 12.7 | 53 | 1-7 | F | 295 |
| SS | 423 | 13.9 | 34.3 | 30.8 | 89.9 | 2.37 | 21.3 | 7.3 | 6 | 1-7 | F | 296 |
| N | 412 | 6.3 | 34.9 | 28.7 | 82.1 | 5.13 | 42.1 | 14.7 | 4 | 14-18 | M | 297 |
| N | 261 | 5.8 | 34.3 | 28.3 | 82.5 | 5.02 | 41.4 | 14.2 | 4 | 8-13 | F | 297 |
| N | 153 | 7.5 | 33.0 | 27.1 | 82.3 | 4.68 | 38.5 | 12.7 | 3 | 8-13 | M | 298 |
| N | 313 | 4.6 | 32.9 | 27.2 | 82.7 | 4.45 | 36.8 | 12.1 | 3 | 8-13 | M | 299 |
| N | 363 | 6.5 | 32.2 | 28.0 | 87.0 | 3.78 | 32.9 | 10.6 | 3 | 1-7 | M | 300 |
| N | 309 | 4.8 | 31.3 | 26.6 | 84.8 | 4.67 | 39.6 | 12.4 | 3 | More than 18 | F | 301 |
| N | 214 | 7.2 | 33.4 | 27.9 | 83.4 | 3.55 | 29.6 | 9.9 | 3 | 14-18 | F | 302 |
| N | 191 | 4.9 | 32.9 | 27.4 | 83.2 | 5.37 | 44.7 | 14.7 | 1 | 14-18 | M | 304 |
| N | 200 | 8.8 | 34.0 | 29.9 | 87.9 | 5.35 | 47.0 | 16.0 | 1 | 14-18 | M | 305 |
| N | 183 | 3.9 | 35.4 | 29.5 | 83.3 | 4.92 | 41.0 | 14.5 | 1 | 8-13 | M | 306 |
| N | 358 | 6.4 | 35.0 | 29.4 | 83.8 | 4.94 | 41.4 | 14.6 | 1 | 8-13 | M | 307 |

| N | | 164 | 7.1 | | 33.5 | | 26.0 | | 77.7 | | 5.30 | | 41.2 | | 13.8 | | 1 | 14-18 | | M | | 308 | |
| --- | --- | --- | --- | --- | --- | --- | --- | --- | --- | --- | --- | --- | --- | --- | --- | --- | --- | --- | --- | --- | --- | --- | --- |
| N | | 130 | 4.0 | 28.7 | | 27.7 | | 96.5 | | 5.49 | | 53.0 | | 15.2 | | 1 | | 14-18 | M | | 309 | |  |
| N | | 323 | 8.4 | 34.5 | | 28.8 | | 83.6 | | 4.51 | | 37.7 | | 13.0 | | 1 | | 8-13 | M | | 310 | |  |
| N | | 27 | 17.9 | 32.4 | | 27.1 | | 83.5 | | 3.40 | | 28.4 | | 9.2 | | 1 | | 8-13 | F | | 311 | |  |
| N | | 516 | 7.4 | 33.7 | | 24.5 | | 72.6 | | 4.49 | | 32.6 | | 11.0 | | 1 | | 14-18 | M | | 312 | |  |
| N | | 506 | 12.5 | 33.5 | | 26.3 | | 78.3 | | 4.11 | | 32.2 | | 10.8 | | 13 | | Less than 1 | M | | 313 | |  |
| N | | 357 | 10.3 | 34.7 | | 26.5 | | 76.5 | | 3.96 | | 30.3 | | 10.5 | | 1 | | Less than 1 | M | | 314 | |  |
| N | | 239 | 8.9 | 32.7 | | 27.2 | | 83.2 | | 4.63 | | 38.5 | | 12.6 | | 3 | | 1-7 | M | | 315 | |  |
| N | | 203 | 38.8 | 33.8 | | 26.7 | | 78.9 | | 4.50 | | 35.5 | | 12.0 | | 4 | | Less than 1 | F | | 316 | |  |
| N | | 181 | 9.0 | 35.6 | | 32.0 | | 90.0 | | 3.50 | | 31.5 | | 11.2 | | 1 | | Less than 1 | F | | 317 | |  |
| N | | 669 | 49.3 | 34.2 | | 36.2 | | 106.0 | | 1.49 | | 15.8 | | 5.4 | | 15 | | 1-7 | M | | 318 | |  |
| N | | 261 | 1.7 | 31.6 | | 26.6 | | 84.2 | | 3.68 | | 31.0 | | 9.8 | | 26 | | 8-13 | M | | 319 | |  |
| N | | 255 | 8.5 | 33.8 | | 25.3 | | 74.9 | | 4.11 | | 30.8 | | 10.4 | | 26 | | Less than 1 | F | | 320 | |  |
| N | | 270 | 10.6 | 31.7 | | 21.5 | | 67.9 | | 4.70 | | 31.9 | | 10.1 | | 13 | | Less than 1 | F | | 321 | |  |
| N | | 295 | 10.1 | 33.2 | | 24.8 | | 74.7 | | 4.07 | | 30.4 | | 10.1 | | 18 | | Less than 1 | F | | 322 | |  |
| N | | 399 | 8.6 | 32.4 | | 25.8 | | 79.6 | | 4.50 | | 35.8 | | 11.6 | | 1 | | Less than 1 | F | | 323 | |  |
| N | | 231 | 19.6 | 30.9 | | 22.7 | | 73.4 | | 4.85 | | 35.6 | | 11.0 | | 9 | | 1-7 | M | | 324 | |  |
| N | | 61 | 5.7 | 32.7 | | 23.4 | | 71.5 | | 5.09 | | 36.4 | | 11.9 | | 3 | | 8-13 | M | | 325 | |  |
| N | | 311 | 7.4 | 35.0 | | 28.3 | | 80.8 | | 4.07 | | 32.9 | | 11.5 | | 1 | | Less than 1 | M | | 326 | |  |
| N | | 349 | 6.0 | 32.3 | | 23.7 | | 73.3 | | 3.93 | | 28.8 | | 9.3 | | 1 | | Less than 1 | F | | 327 | |  |
| N | | 432 | 10.9 | 30.9 | | 21.6 | | 70.0 | | 5.23 | | 36.6 | | 11.3 | | 13 | | Less than 1 | M | | 328 | |  |
| N | | 99 | 8.5 | 33.5 | | 27.9 | | 83.3 | | 4.37 | | 36.4 | | 12.2 | | 21 | | 1-7 | M | | 329 | |  |
| N | | 63 | 6.7 | 32.3 | | 23.9 | | 73.9 | | 3.56 | | 26.3 | | 8.5 | | 13 | | Less than 1 | M | | 330 | |  |
| N | | 414 | 10.7 | 31.7 | | 24.8 | | 78.2 | | 4.36 | | 34.1 | | 10.8 | | 3 | | Less than 1 | M | | 331 | |  |
| N | | 64 | 12.2 | 34.0 | | 23.3 | | 68.7 | | 4.50 | | 30.9 | | 10.5 | | 55 | | Less than 1 | F | | 332 | |  |
| N | | 199 | 11.1 | 34.4 | | 28.9 | | 83.9 | | 1.80 | | 15.1 | | 5.2 | | 4 | | Less than 1 | F | | 333 | |  |
| N | | 398 | 13.3 | 34.6 | | 24.5 | | 71.0 | | 4.93 | | 35.1 | | 12.1 | | 4 | | Less than 1 | M | | 334 | |  |
| N | | 322 | 12.2 | 32.5 | | 23.9 | | 73.5 | | 5.06 | | 37.2 | | 12.1 | | 6 | | 8-13 | M | | 335 | |  |
| N | | 385 | 10.5 | 31.0 | | 28.4 | | 91.5 | | 4.69 | | 42.9 | | 13.3 | | 1 | | 14-18 | F | | 336 | |  |
| N | | 217 | 9.4 | 29.9 | | 24.7 | | 82.7 | | 5.54 | | 45.8 | | 13.7 | | 3 | | 8-13 | | F | 337 | |  |
| N | | 319 | 6.6 | 31.0 | | 26.0 | | 84.0 | | 4.19 | | 35.2 | | 10.9 | | 1 | | Less than 1 | | F | 338 | |  |
| N | | 312 | 4.6 | 30.2 | | 25.3 | | 83.6 | | 4.63 | | 38.7 | | 11.7 | | 1 | | 1-7 | | F | 339 | |  |
| N | | 245 | 4.9 | 30.4 | | 25.5 | | 84.0 | | 4.74 | | 39.8 | | 12.1 | | 1 | | 8-13 | | F | 340 | |  |
| N | | 206 | 6.3 | 31.5 | | 25.8 | | 81.7 | | 4.93 | | 40.3 | | 12.7 | | 1 | | 1-7 | M | | 341 | |  |
| N | | 293 | 5.3 | 31.6 | | 26.5 | | 84.0 | | 4.94 | | 41.5 | | 13.1 | | 1 | | 8-13 | F | | 342 | |  |
| N | | 409 | 1.5 | 32.4 | | 31.6 | | 97.5 | | 4.81 | | 46.9 | | 15.2 | | 1 | | 8-13 | M | | 343 | |  |
| N | | 303 | 5.5 | 31.5 | | 29.0 | | 92.1 | | 4.41 | | 40.6 | | 12.8 | | 3 | | 8-13 | F | | 344 | |  |
| N | | 254 | 4.6 | 29.1 | | 23.0 | | 79.2 | | 4.86 | | 38.5 | | 11.2 | | 1 | | 8-13 | F | | 345 | |  |
| N | | 281 | 4.2 | 33.1 | | 29.6 | | 89.6 | | 5.27 | | 47.2 | | 12.0 | | 3 | | 14-18 | M | | 346 | |  |
| N | | 222 | 6.6 | 32.8 | | 25.7 | | 78.2 | | 5.69 | | 44.5 | | 13.0 | | 1 | | 14-18 | M | | 347 | |  |
| N | | 213 | 1.7 | 32.5 | | 28.1 | | 86.3 | | 5.34 | | 46.1 | | 12.8 | | 1 | | 8-13 | M | | 348 | |  |
| N | | 311 | 8.1 | 28.8 | | 26.0 | | 90.4 | | 4.57 | | 41.3 | | 12.3 | | 2 | | 8-13 | F | | 349 | |  |
| N | | 265 | 2.0 | 32.3 | | 29.9 | | 92.4 | | 4.35 | | 40.2 | | 12.1 | | 1 | | 14-18 | F | | 350 | |  |
| N | 265 | | 2.0 | 32.3 | | 29.9 | | 92.4 | | | 4.35 | | 40.2 | 13.0 | | 1 | | 14-18 | | F | | 350 | |
| N | 120 | | 21.6 | 32.4 | | 23.1 | | 71.5 | | | 4.45 | | 31.8 | 11.8 | | 1 | | Less than 1 | | M | | 351 | |
| N | 179 | | 14.4 | 34.2 | | 27.0 | | 78.8 | | | 4.19 | | 33.0 | 12.1 | | 6 | | 8-13 | | F | | 352 | |

| N | 237 | 7.4 | 32.6 | 25.9 | 79.4 | 5.06 | 40.2 | 13.1 | 32 | 14-18 | M | 353 |
| --- | --- | --- | --- | --- | --- | --- | --- | --- | --- | --- | --- | --- |
| N | 349 | 5.1 | 30.9 | 19.5 | 63.2 | 5.73 | 36.2 | 11.2 | 32 | 1-7 | M | 354 |
| N | 364 | 6.6 | 32.7 | 25.2 | 77.0 | 4.92 | 37.9 | 12.4 | 32 | 1-7 | F | 355 |
| N | 120 | 1.7 | 33.7 | 28.6 | 85.1 | 4.82 | 41.0 | 13.8 | 32 | 14-18 | F | 356 |
| N | 193 | 6.0 | 33.6 | 28.8 | 85.8 | 4.23 | 36.3 | 12.2 | 32 | 8-13 | F | 357 |
| N | 246 | 4.3 | 34.9 | 26.5 | 76.1 | 4.60 | 35.0 | 12.2 | 40 | 8-13 | F | 358 |
| N | 267 | 4.9 | 32.9 | 27.6 | 84.0 | 4.45 | 37.4 | 12.3 | 40 | 14-18 | F | 359 |
| N | 263 | 5.3 | 32.9 | 20.9 | 63.7 | 4.87 | 31.0 | 10.2 | 48 | 8-13 | F | 360 |
| N | 98 | 4.8 | 32.9 | 27.8 | 84.5 | 5.36 | 45.3 | 14.9 | 48 | 14-18 | F | 361 |
| N | 176 | 4.1 | 32.4 | 26.0 | 80.2 | 5.31 | 42.2 | 13.8 | 48 | 8-13 | M | 362 |

| N | 306 | 5.0 | 34.7 | 30.0 | 86.5 | 4.23 | 36.6 | 12.7 | 3 | 14-18 | F | | 363 |
| --- | --- | --- | --- | --- | --- | --- | --- | --- | --- | --- | --- | --- | --- |
| N | 216 | 4.3 | 31.0 | 27.7 | 89.3 | 4.41 | 39.4 | 12.2 | 3 | 8-13 | F | | 364 |
| N | 272 | 6.5 | 30.7 | 29.5 | 96.3 | 4.91 | 47.3 | 14.5 | 3 | 14-18 | M | | 365 |
| N | 197 | 6.2 | 29.8 | 24.8 | 83.2 | 4.76 | 39.6 | 11.8 | 4 | 1-7 | M | | 366 |
| N | 327 | 6.0 | 31.8 | 27.0 | 84.9 | 4.63 | 39.3 | 12.5 | 4 | 14-18 | M | | 367 |
| N | 225 | 5.1 | 29.2 | 25.8 | 88.2 | 4.23 | 37.3 | 10.9 | 1 | 14-18 | F | 368 | |
| N | 116 | 14.2 | 30.7 | 27.2 | 88.7 | 4.15 | 36.8 | 13.9 | 3 | 1-7 | M | 369 | |
| N | 102 | 8.6 | 31.2 | 27.6 | 88.6 | 3.87 | 34.3 | 10.7 | 4 | 1-7 | M | 370 | |
| N | 226 | 5.5 | 34.8 | 31.7 | 91.1 | 4.48 | 40.8 | 14.2 | 3 | 14-18 | F | 371 | |
| N | 264 | 9.3 | 33.7 | 27.8 | 82.5 | 5.03 | 41.5 | 14.0 | 4 | 8-13 | M | 372 | |
| N | 94 | 2.3 | 29.0 | 20.6 | 71.1 | 4.18 | 29.7 | 8.6 | 4 | 8-13 | F | 373 | |
| N | 247 | 7.1 | 34.1 | 29.9 | 87.5 | 4.89 | 42.8 | 14.6 | 4 | 14-18 | F | 374 | |
| N | 143 | 4.0 | 36.2 | 34.0 | 94.1 | 3.88 | 36.5 | 13.2 | 3 | more than 18 | F | 375 | |
| N | 572 | 32.4 | 34.0 | 31.2 | 91.8 | 2.31 | 21.2 | 7.2 | 3 | 14-18 | M | | 374 |
| N | 245 | 5.0 | 30.5 | 26.6 | 87.1 | 4.33 | 37.7 | 11.5 | 3 | 14-18 | F | | 375 |
| N | 158 | 4.3 | 33.2 | 29.2 | 88.2 | 4.65 | 41.0 | 13.6 | 3 | 8-13 | F | | 376 |
| N | 102 | 3.7 | 30.5 | 20.4 | 66.8 | 5.64 | 37.7 | 11.5 | 3 | 14-18 | M | | 377 |
| N | 192 | 6.2 | 32.6 | 27.8 | 85.4 | 4.17 | 35.6 | 11.6 | 3 | 14-18 | F | | 378 |
| N | 244 | 5.1 | 34.2 | 30.6 | 89.3 | 4.12 | 36.8 | 12.6 | 3 | 14-18 | F | | 379 |
| N | 101 | 7.8 | 35.1 | 28.2 | 80.3 | 5.18 | 41.6 | 14.6 | 4 | 8-13 | F | | 380 |
| N | 368 | 5.8 | 31.6 | 27.5 | 87.0 | 4.55 | 39.6 | 12.5 | 4 | 14-18 | F | | 381 |
| N | 386 | 8.1 | 33.2 | 25.2 | 75.9 | 4.68 | 35.5 | 11.8 | 1 | 1-7 | F | | 382 |
| N | 208 | 8.3 | 34.5 | 27.6 | 79.9 | 4.53 | 36.2 | 12.5 | 3 | 1-7 | M | | 383 |
| N | 338 | 7.7 | 34.3 | 29.5 | 85.9 | 3.70 | 31.8 | 10.9 | 3 | 14-18 | F | | 384 |
| N | 311 | 7.4 | 32.7 | 25.4 | 77.6 | 5.08 | 39.4 | 12.9 | 3 | 8-13 | M | | 385 |
| N | 224 | 10.8 | 33.4 | 27.4 | 82.1 | 3.68 | 30.2 | 10.1 | 3 | 8-13 | F | | 386 |
| N | 235 | 4.1 | 34.0 | 27.7 | 81.6 | 4.40 | 35.9 | 12.2 | 4 | 1-7 | M | | 387 |
| N | 235 | 9.4 | 32.0 | 23.5 | 73.4 | 5.07 | 37.2 | 11.9 | 3 | 14-18 | M | | 388 |
| N | 180 | 7.1 | 34.3 | 27.2 | 79.5 | 3.56 | 28.3 | 9.7 | 1 | 8-13 | F | | 389 |
| N | 188 | 3.6 | 34.5 | 27.9 | 80.9 | 4.55 | 36.8 | 12.7 | 1 | 8-13 | M | | 390 |
| N | 162 | 7.1 | 33.1 | 29.9 | 90.3 | 4.75 | 42.9 | 14.2 | 8 | 1-7 | 13 | | 391 |
| N | 192 | 1.1 | 32.3 | 29.0 | 89.8 | 4.86 | 43.7 | 14.1 | 13 | 8-13 | F | | 392 |
| N | 272 | 2.4 | 33.1 | 30.7 | 92.9 | 4.62 | 42.9 | 14.2 | 16 | 8-13 | F | | 393 |
| N | 255 | 2.9 | 31.6 | 26.8 | 84.6 | 5.27 | 44.6 | 14.1 | 6 | 14-18 | F | | 394 |
| N | 203 | 3.4 | 33.3 | 28.5 | 85.4 | 4.85 | 41.4 | 13.8 | 6 | 14-18 | F | | 395 |
| N | 145 | 2.5 | 33.0 | 29.4 | 89.0 | 4.73 | 42.1 | 13.9 | 24 | 8-13 | F | | 396 |
| N | 259 | 2.9 | 32.5 | 29.6 | 91.3 | 4.59 | 41.9 | 13.6 | 25 | 14-18 | F | | 397 |
| SS | 504 | 1.7 | 31.4 | 29.5 | 93.9 | 2.61 | 24.5 | 7.7 | 6 | 8-13 | M | | 398 |
| N | 320 | 2.1 | 33.3 | 28.5 | 85.4 | 4.32 | 36.9 | 12.3 | 6 | 14-18 | M | | 399 |
| N | 227 | 2.8 | 34.6 | 27.8 | 80.3 | 4.49 | 35.8 | 12.4 | 26 | 1-7 | M | | 400 |

| Key | |
| --- | --- |
| The Tribes code | |
| ZAGAWA | 1 |
| TAAYSHA | 2 |
| FUR | 3 |
| TONGOUR | 4 |
| HAWARA | 5 |
| HOUSA | 6 |
| DAJO | 7 |
| MOSABAAT | 8 |
| MEDOB | 9 |
| KENANA | 10 |
| MASALEET | 11 |
| AWLAD MANA | 12 |
| BARTEY | 13 |
| NUBA | 14 |
| ZIADYA | 15 |
| FALLATA | 16 |
| MEMA | 17 |
| HABANEYA | 18 |
| SHOYHAT | 19 |
| KAWAHLA | 20 |
| KENEEN | 21 |
| ETAFAAT | 22 |
| BARNO | 23 |
| TAMA | 24 |
| BRAGO | 25 |
| BNI HSSEN | 26 |
| MARARET | 27 |
| GEMER | 28 |
| REZEGAT | 29 |
| BNI HLBA | 30 |
| SLEHAB | 31 |
| KROBAT | 32 |
| MESERIA | 33 |
| AWLD RASHED | 34 |
| ARAB BASHER | 35 |
| HEMEDIA | 36 |
| BNI OMRAN | 37 |
| GALEIA | 38 |
| DADENGA | 39 |
| GAWAMA | 40 |
| SLAMAAT | 41 |
| SHYGIA | 42 |
| DANGLA | 43 |
| MAALYA | 44 |
| SHRAFA | 45 |
| HAMR | 46 |
| TKAREER | 47 |
| ASERAA | 48 |
| AWLAD ALREEF | 49 |
| KHOZAM | 50 |
| QURAN | 51 |
| BNI MANSOOR | 52 |
| BAZAA | 53 |
| BAGRMA | 54 |
| DAR HAMID | 55 |
| WAHEIA | 56 |
| MANASEER | 57 |
| KALEMBO | 58 |
| KTENGA | 59 |
