# Supplementary material for: Prevalence of sickle cell disease and sickle cell trait among children admitted to Al Fashir Teaching Hospital North Darfur State, Sudan
Source: BMC Res Notes. 2019 Oct 16;12:659. doi: 10.1186/s13104-019-4682-5 (PMC6796395; doi:10.1186/s13104-019-4682-5)
Supplement: Supplementary file 2 — Additional file 2. Examples for the study populations families informed consents. [file 13104_2019_4682_MOESM2_ESM.docx]

**Table S1: Frequency of tribes contribute in the study.**

| **Tribes** | **Frequency** | **Percentages (%)** |
| --- | --- | --- |
| Zagawa | 97 | 24.3 |
| Fur | 74 | 18.5 |
| Bartey | 34 | 8.5 |
| Tongour | 31 | 7.8 |
| Housa | 22 | 5.5 |
| Ziadya | 17 | 4.3 |
| Fallata | 14 | 3.5 |
| Kenen | 13 | 3.3 |
| Tama | 9 | 2.3 |
| Hawara | 7 | 1.8 |
| Taaysha | 5 | 1.3 |
| Barno | 5 | 1.3 |
| Krobat | 5 | 1.3 |
| Meserria | 5 | 1.3 |
| Gawama | 5 | 1.3 |
| Bazaa | 5 | 1.3 |
| Medob | 4 | 1 |
| Awlad Mana | 4 | 1 |
| Bni Hssen | 4 | 1 |
| Aseraa | 4 | 1 |
| Quran | 4 | 1 |
| Mema | 3 | 0.75 |
| Habaneya | 3 | 0.75 |
| Bargo | 3 | 0.75 |
| Mararet | 3 | 0.75 |
| Kenana | 2 | 0.5 |
| Masaleet | 2 | 0.5 |
| Rezagat | 2 | 0.5 |
| Sharfa | 2 | 0.5 |
| Mosabaat | 1 | 0.25 |
| Shoyhat | 1 | 0.25 |
| Kawahla | 1 | 0.25 |
| Etafaat | 1 | 0.25 |
| Gemer | 1 | 0.25 |
| Awld Rashed | 1 | 0.25 |
| Arab Basher | 1 | 0.25 |
| Awlad Alreef | 1 | 0.25 |
| Khozam | 1 | 0.25 |
| Dar hamid | 1 | 0.25 |
| Kalembo | 1 | 0.25 |
| Ktenga | 1 | 0.25 |
| Total | 400 | 100 |
